# Comparative genomic analysis of mycobacteriophage Tweety: evolutionary insights and construction of compatible site-specific integration vectors for mycobacteria

**DOI:** 10.1099/mic.0.2007/008904-0

**Published:** 2007-08

**Authors:** Thuy T. Pham, Deborah Jacobs-Sera, Marisa L. Pedulla, Roger W. Hendrix, Graham F. Hatfull

**Affiliations:** 1 Department of Biological Sciences and Pittsburgh Bacteriophage Institute, University of Pittsburgh, Pittsburgh, PA 15260, USA; 2 Department of Biology, Montana Tech, University of Montana, Butte, MT 59701, USA

## Abstract

Mycobacteriophage Tweety is a newly isolated phage of *Mycobacterium smegmatis*. It has a viral morphology with an isometric head and a long flexible tail, and forms turbid plaques from which stable lysogens can be isolated. The Tweety genome is 58 692 bp in length, contains 109 protein-coding genes, and shows significant but interrupted nucleotide sequence similarity with the previously described mycobacteriophages Llij, PMC and Che8. However, overall the genome possesses mosaic architecture, with gene products being related to other mycobacteriophages such as Che9d, Omega and Corndog. A gene encoding an integrase of the tyrosine-recombinase family is located close to the centre of the genome, and a putative *attP* site has been identified within a short intergenic region immediately upstream of *int*. This Tweety *attP*–*int* cassette was used to construct a new set of integration-proficient plasmid vectors that efficiently transform both fast- and slow-growing mycobacteria through plasmid integration at a chromosomal locus containing a tRNA^Lys^ gene. These vectors are maintained well in the absence of selection and are completely compatible with integration vectors derived from mycobacteriophage L5, enabling the simple construction of complex recombinants with genes integrated simultaneously at different chromosomal positions.

## INTRODUCTION

The isolation and comparative genomic analysis of 30 mycobacteriophages (viruses that infect mycobacterial hosts) reveals them to have high genetic diversity and to typically contain genomes that are genetic mosaics with modules shared with other phage genomes (Hatfull *et al.*, 2006; Pedulla *et al.*, 2003). Nucleotide sequence comparison of these genomes shows that while they are overall highly diverse, there are several smaller clusters within the group with genomes that are more similar to each other than to other mycobacteriophage genomes. Similar clusters were revealed through a metaproteomic analysis in which all 3350 putative protein products were organized into related ‘phamilies’ and the genomes compared according to the presence or absence of phamily members (Hatfull *et al.*, 2006). The availability of groups of closely related phages in the context of a larger, more diverse, group significantly enhances the power of comparative genomic analyses. In particular, comparisons among closely related sequences have the potential to reveal the nature of individual mutational steps of phage evolution, unconfounded by multiple overlapping events.

Characterization of mycobacteriophage genomes not only provides insights into viral diversity and evolution but also offers a large, diverse and complex toolbox from which a variety of applications useful for mycobacterial genetics can be derived. A recent example is the identification of mycobacteriophage genes encoding recombination functions related to RecE and RecT which, while rare among mycobacteriophages, are both found in phage Che9c (van Kessel & Hatfull, 2007). These have been utilized to develop a recombineering system to facilitate the construction of gene-replacement mutants by allelic exchange in both *Mycobacterium smegmatis* and *Mycobacterium tuberculosis* (van Kessel & Hatfull, 2007). Other examples include the use of phage immunity loci as genetically selectable markers (Donnelly-Wu *et al.*, 1993; Jain & Hatfull, 2000), regulated gene expression systems (Brown *et al.*, 1997), and exploitation of phage integration systems (Lee *et al.*, 1991).

The construction of integration-proficient plasmid vectors based on the integration system of mycobacteriophage L5 enables the simple insertion of genes into the chromosomes of both fast- and slow-growing mycobacteria (Lee *et al.*, 1991; Stover *et al.*, 1991) and similar vectors based on other phages such as Ms6 have been described previously (Freitas-Vieira *et al.*, 1998). Provided that the phage-encoded recombination directionality factor (RDF) (Lewis & Hatfull, 2001) is not present in these vectors then the integrated DNAs are more stably maintained in the absence of selection than extrachromosomal plasmid vectors; however, excision-independent integrase-mediated excisive recombination can lead to plasmid loss and accumulation of excised derivatives, especially when the integrated sequences express products deleterious to growth of the recombinant (Springer *et al.*, 2001). These events can be avoided by using transient expression of integrase to construct recombinants such that the *int* gene is not present in the stably transformed strains (Hatfull, 2004; Peña *et al.*, 1997). Introduction of the L5 excise (gene *36*) into integrated recombinants leads to efficient integrase-mediated excision (Lewis & Hatfull, 2000) and this has been exploited to determine whether genes are essential for mycobacterial growth (Parish *et al.*, 2001) and to switch integrated plasmid copies (Pashley & Parish, 2003).

A primary benefit of these integration-proficient vector systems is that they enable the construction of single-copy recombinants that avoid the phenotypic effects of multicopy recombinants, including phage and drug resistance (Banerjee *et al.*, 1994; Barsom & Hatfull, 1996). However, there are often genetic applications that require the insertion of more than one element into the chromosome and methods have been described to piggy-back multiple insertions using L5 integration vectors (Saviola & Bishai, 2004), to manipulate Ms6 vectors to confer integration at different chromosomal loci (Vultos *et al.*, 2006), or to use alternative integration systems such as those derived from serine-integrases *φ*Rv1 and Bxb1 (Bibb *et al.*, 2005; Kim *et al.*, 2003). However, these have significant limitations, including reduced frequency, limited strain utilization, or, in the case of the serine-integrases, interruption of chromosomal genes (Kim *et al.*, 2003; Ojha *et al.*, 2005). There is thus a need for additional integration-proficient vectors that are fully compatible with other vector systems.

In this paper we describe the isolation and genomic characterization of mycobacteriophage Tweety and the development of integration-proficient plasmids carrying the Tweety *attP*–*int* region that efficiently transform both fast- and slow-growing mycobacteria. These Tweety-derived vectors integrate at a tRNA^Lys^ gene as distinct from the tRNA^Gly^ chromosomal locus used by L5-derived vectors and are fully compatible, such that co-transformants with both integrating vector systems can be recovered from a single electroporation. *M. smegmatis* recombinants derived by Tweety-mediated integration are more stably maintained than recombinants derived using L5 integration-proficient vectors in the presence of their cognate integrases and should prove to be useful additions to the arsenal of tools available for mycobacterial genetic manipulation.

## METHODS

### Bacterial strains.

*Mycobacterium smegmatis* mc^2^155 and *Mycobacterium bovis* bacille Calmette–Guérin (BCG) have been described previously (Jacobs *et al.*, 1991; Snapper *et al.*, 1990); electrocompetent cells were prepared as described previously (Bibb & Hatfull, 2002) and transformed using 0.05–1 μg DNA. Media were supplemented with carbenicillin (50 μg ml^−1^), cycloheximide (10 μg ml^−1^), kanamycin (20 μg ml^−1^), hygromycin (50 μg ml^−1^) or gentamicin (10 μg ml^−1^) as required.

### Phage isolation and genome sequencing.

Tweety was isolated from a moist soil sample from a lawn in the Oakland district near the University of Pittsburgh (PA, USA) . Tweety was plaque purified and sequenced using a shotgun approach as described previously (Pedulla *et al.*, 2003; Sarkis & Hatfull, 1998). The GenBank accession number is EF536069.

### Plasmids and DNA.

Plasmid pMH94 is an L5 integration-proficient vector that has been described previously (Lee *et al.*, 1991). Plasmids pJV39 and pJV44 were kind gifts from Julia van Kessel, University of Pittsburgh. Plasmid pJV39 is similar to pMH94 but confers hygromycin resistance (Hyg^R^) instead of kanamycin resistance (Kan^R^). Plasmids pTTP1A and pTTP1B were constructed as follows. Two primers with *Xho*I restriction sites were designed and used to amplify the *attP* and *int* region from Tweety genomic DNA. This 1.7 kb fragment was inserted by blunt-end cloning into vector pMOSBlue. A clone containing Tweety *attP* and *int* was identified and digested with *Xho*I, and the fragment was subcloned into *Sal*I-digested pMH94. Both pTTP1A and pTTP1B contain the Tweety *attP* and *int*, *oriE*, and kanamycin- and ampicillin-resistance genes. Plasmids pTTP1A and pTTP1B differ in regard to the orientation of the *attP–int* region with respect to the plasmid backbone. DNA manipulations and agarose gel electrophoresis were as described by Sambrook *et al.* (1989).

### PCR assays.

Site-specific integration between the homologous sequences of Tweety *attP* and *M. smegmatis attB* was confirmed using PCR assays. Transformants were prepared for colony PCR by suspending in 200 μl H_2_O, vortexing 20 times, and heating at 95 °C for 5 min. Approximately 5 μl of the colony mix was used along with *Pfu* polymerase (Stratagene), dNTPs (10 mM) and 5 % (v/v) DMSO. The four primers used to amplify the *attL* and *attR* regions of the recombinant chromosome were TTP1a (5′-CAGTCACGACGTTGTAAAACGACGG-3′), TTP1b (5′-GTCACCGAAAGGCGTGCCCTTGTC-3′), TTP1d (5′-GACCGCTTCAAGAGCGAGCAGTAC-3′) and TTP1e (5′-TCCCGTTGAATATGGCTCATAACACCC-3′). PCR products were analysed by gel electrophoresis.

### Plasmid stability.

Transformants of *M. smegmatis* derived from pTTP1A and pTTP1B were inoculated into Middlebrook 7H10 medium containing ADC [albumin (5 g l^−1^), dextrose (2 g l^−1^), NaCl (0.85 g l^−1^)], Tween 80 (0.05 %) and kanamycin (20 μg ml^−1^) and grown to saturation. Cultures were diluted 1 : 10 000 into antibiotic-free medium and allowed to grow back to saturation (Lee *et al.*, 1991). The cultures were repeatedly diluted and grown for a total of approximately 35 generations. Cell samples were then plated for single colonies on solid 7H10/ADC medium in the presence and absence of kanamycin to determine the proportion of antibiotic-resistant colonies. 

### Electron microscopy.

A suspension of CsCl-purified virions was applied to a sample grid with a carbon-coated nitrocellulose film, stained with 2 % uranyl acetate, and examined in a FEI Morgagni 268 transmission electron microscope equipped with an AMT digital camera system.

## RESULTS

### Isolation and genomic sequencing of mycobacteriophage Tweety

**Fig. 1. f1:**
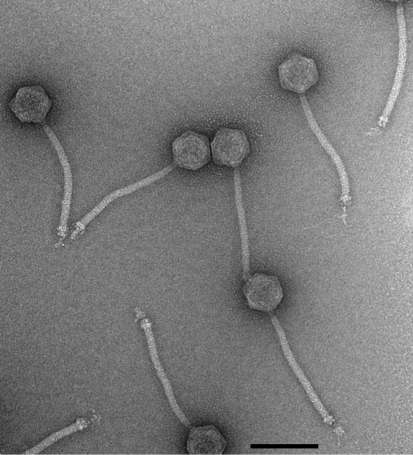
Virion morphology of Tweety. Electron micrograph of mycobacteriophage Tweety particles. Scale bar, 100 nm.

Mycobacteriophage Tweety was isolated from a soil sample from the Oakland district of Pittsburgh, and was identified (without amplification) as a p.f.u. on a lawn of *M. smegmatis* mc^2^155. Following plaque purification, high-titre stocks were prepared and Tweety virions were examined by electron microscopy. Tweety particles have a morphology typical of the Siphoviridae, with an isometric head approximately 60 nm in diameter and a flexible tail approximately 175 nm long (Fig. 1); this morphology is the most common one found among mycobacteriophages (Hatfull *et al.*, 2006). A small, possibly double-layered, baseplate structure is visible at the tip of the tail, with some apparently flexible fibrous structures extending beyond it. Tweety forms lightly turbid plaques on lawns of *M. smegmatis*, a plaque morphology that is extremely common among mycobacteriophages.

**Fig. 2. f2:**
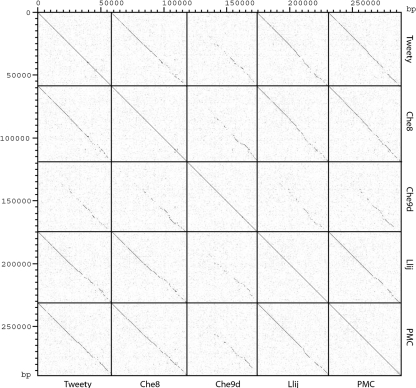
Nucleotide sequence comparisons of the Tweety, Che8, Che9d, Llij and PMC genomes. The extent of DNA sequence similarity among these mycobacteriophage genomes is illustrated in a Dotter plot using a sliding window of 25 bp (Sonnhammer & Durbin, 1995).

Preliminary restriction analysis of Tweety dsDNA from virions indicated that it is distinct from all previously characterized mycobacteriophages, and its complete genomic sequence was determined using a shotgun strategy. Tweety virion DNA is 58 692 bp in length; it has unique ends with 10 base, single-stranded cohesive 3′-extensions (left end; 3′-GCCTTCCGCG). The Tweety genome is 61.7 mol% G+C, similar to other mycobacteriophage and mycobacterial genomes. Nucleotide sequence comparison with the 30 previously sequenced mycobacteriophage genomes revealed significant sequence similarity with mycobacteriophages Che8, Llij, PMC and, to a lesser degree, Che9d (Fig. 2). The extent of sequence similarity between Tweety and Che8, Llij and PMC appears to be highest in the leftmost parts of these genomes, while being weaker and discontinuous towards the rightmost parts.

### Organization of the Tweety genome

**Fig. 3. f3:**
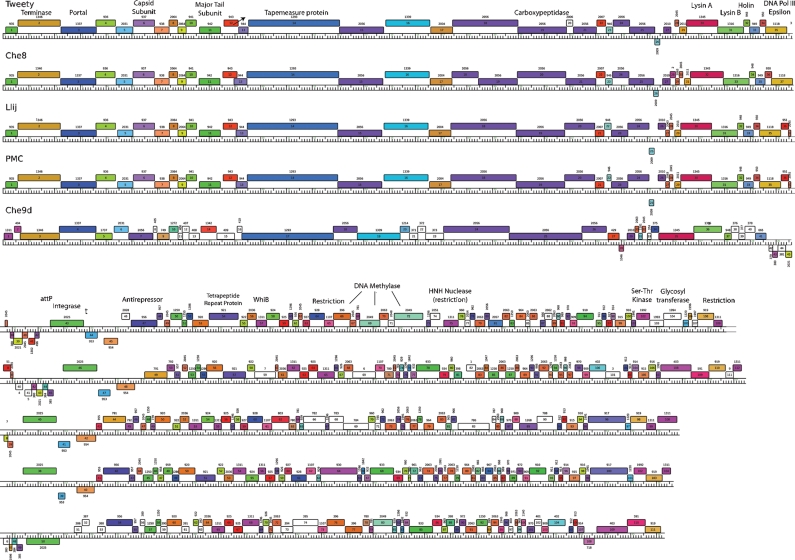
Map of the Tweety genome and comparison to maps of Che8, Llij, PMC and Che9d. Genomes are represented by horizontal lines with putative genes shown as boxes above (transcribed rightwards) or below (transcribed leftwards) each genome; the number of each gene is shown within each box. The diagonal arrow indicates a programmed translational frameshift between Tweety genes *12* and *13*. All genes have been assorted into phamilies (Phams) of related sequences using the computer program ‘Phamerator’ (S. Cresawn, R. W. Hendrix & G. F. Hatfull, unpublished data); the phamily number is displayed above each gene and the boxes colour-coordinated accordingly. Note that the Pham numbers differ from those described previously (Hatfull *et al.*, 2006). Putative gene functions are noted. (A larger version of this figure is available as supplementary data with the online version of this paper.)

Analysis of the Tweety genome reveals 109 potential ORFs (Table 1), all except eight of which are transcribed in the rightwards direction (Fig. 3). The overall genome organization shares similarities with other mycobacteriophages such as PMC, Llij and Che8 and differs from phages such as L5, D29 and their near relatives, in which genes in the right half of the genome are transcribed leftwards (Ford *et al.*, 1998; Hatfull & Sarkis, 1993; Hatfull *et al.*, 2006; Pedulla *et al.*, 2003). Most of the Tweety genome is utilized as protein-coding regions, although there are small non-coding regions between gene *109* and the right terminus, and between genes *42* and *43*, and genes *44* and *45*. We have not identified any tRNA, transfer-messenger RNA (tmRNA) or other small RNA genes. An integrase gene (*43*) of the tyrosine recombinase family lies close to the centre of the genome, and the left arm (genes *1*–*42*) is very similar in organization and sequence to the corresponding parts of the Che8, Llij and PMC genomes (Fig. 3), with the main differences at the right end of the left arm. A putative stem–loop terminator for rightwards transcription is positioned at coordinates 33 784–33 827 immediately following the integrase gene.

### The Tweety genomic left arm: virion structure and assembly genes

Many of the Tweety left arm genes are probably involved in virion structure and assembly, and genes *2*, *3*, *11* and *14* encode putative terminase, portal, major tail subunit and tapemeasure functions respectively, based on sequence similarity to proteins with established functions. Genes *15*, *18*, *19*, *21*, *24* and *25* may all encode minor tail proteins, and we note that the gp19 sequence suggests a carboxypeptidase function, as seen also in several other mycobacteriophage genomes. The two ORFs (*12* and *13*) between the major tail subunit (*11*) and tapemeasure genes (*14*) are arranged such as to express the product of gene *12* (gp12) and a larger protein putatively generated via a translational −2 frameshift approximately 50 bp from the end of gene *12*. By analogy with phage lambda, the gp12 and gp12/13 products are probably involved as chaperones in tail assembly; the programmed frameshift is one of the best-conserved features of dsDNA tailed phages (Xu *et al.*, 2004). The tapemeasure gene is so named because the size of the encoded protein determines the length of the tail (Katsura & Hendrix, 1984; Pedulla *et al.*, 2003). In most cases the proportionality constant relating the two is 0.15 nm tail length per amino acid of tapemeasure protein, corresponding to an *α*-helical structure for the tapemeasure protein. The measured length of the Tweety tail is 175 nm (above), and the 1176 amino acids of the tapemeasure protein would make an *α*-helix of about 176 nm, agreeing very closely with prediction.

The major capsid subunit is likely to be encoded by gene *6*, since we previously showed (unpublished observations) that the Che8 major capsid subunit is Che8 gp6, which is 99 % identical to Tweety gp6. When the sequence databases were searched with the Tweety gp6 sequence using the psi-blast algorithm, more than 100 phage capsid proteins were found, most with very low levels of similarity. Interestingly, after the near-perfect matches of Llij, PMC and Che8, the best matches are to the major capsid proteins of *Escherichia coli* phage T7 and its relatives, with some other mycobacteriophage capsid proteins farther down the list. The Tweety lysis genes (*30*–*32*) are located at the right end of the left arm and encode lysin A (gp30), lysin B (gp31) and holin (gp32) functions respectively. Tweety gp35 has weak but significant similarity (25 % identity, *E*-value, 10^−5^) to a putative DNA polymerase III ε subunit of *Xanthomonas* phage OP1, and the position of a DNA metabolism gene in the left arm is an unusual feature (also found in phages Che8 and Llij). Mycobacteriophage Cjw1 encodes a homologue of Tweety gp35 (Cjw1 gp115), although in this genome it is located at the right end of the right arm (Pedulla *et al.*, 2003).

The Tweety left arm encodes seven proteins (gp15, gp18, gp19, gp20, gp21, gp24 and gp25) that are all part of an extremely large phamily of minor tail proteins that have complex sequence relationships. Tweety gp18 is nearly identical throughout its entire length to Llij gp18 and PMC gp18, but the similar gene in Che8 encodes two proteins gp18 and gp19. A notable departure of the Tweety left arm from its Che8, Llij and PMC relatives is the apparent splitting of the Llij *20*, Che8 *21* and PMC *20* into Tweety genes *20* and *21* (Fig. 3). The DNA sequences of these genes are very closely related although Tweety contains a 1 base deletion at codon 66 that shortens the ORF (see Supplementary Fig. S1, available with the online version of this paper); Tweety gene *21* corresponds to the 3′ end of this segment, although it has a somewhat poor ribosome-binding site and it is uncertain whether it is likely to be expressed. The deletion does not appear to result from a sequencing error (Supplementary Fig. S2) and thus probably corresponds to a genomic change with specific biological consequences for virion particles. We note that a similar single-base deletion in the side tail fibre gene of phage lambda has a specific effect on adsorption to *E. coli* (Hendrix & Duda, 1992) and these may thus reflect the types of mutations that fuel the high degree of variation seen among phage tail fibre proteins (Desplats & Krisch, 2003; Leiman *et al.*, 2006).

### The Tweety genomic right arm

The right arm genes (*44*–*109*) are organized distinctly differently from those of phages Che8, Llij and PMC (Fig. 3) and show evident mosaicism, with numerous insertions and deletions, and many genes related to others dispersed throughout other mycobacteriophage genomes. Only few functions of these right arm genes can be predicted, although these include three possible restriction endonucleases (*65*, *75* and *109*) and three probable DNA methylases (*66*, *69* and *72*). The product of gene *47* is similar to proteins with antirepressor activities, although the immunity functions of Tweety or PMC (which carries a homologue of this protein) have yet to be characterized. We note, however, that gp57 is related to WhiB-family transcriptional regulators, and these are quite common among mycobacteriophage genomes. Tweety also encodes an apparent glycosyl transferase (gp104), a function that has been seen occasionally in other phage genomes, though none of these is a member of the sequence family represented by Tweety gp104. The specific role in the Tweety life cycle is unknown, but since this class of enzymes is associated with modifications of both bacterial cell walls and DNA, it could be involved either in phage exclusion or in protection from restriction. Tweety gp102 has weak sequence similarity to parts of bacterial serine/threonine protein kinases.

### Tweety gp54: a protein with multiple tetrapeptide repeats

**Fig. 4. f4:**
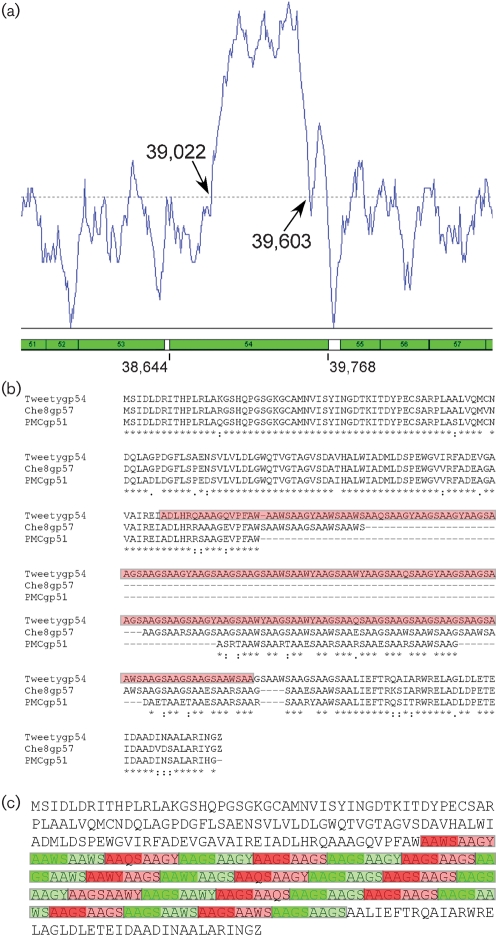
An unusual repeated sequence within Tweety gene *54*. (a) Plot of mol% G+C across the Tweety genome, revealing a region of very high mol% G+C within gene *54*. The approximate positions where changes in mol% G+C occur are indicated. (b) Alignment of Tweety gp54, Che8 gp57 and PMC gp51, with amino acid identities shown by asterisks. Conserved substitutions are indicated by colons, and semi-conserved substitutions by periods. The red box indicates the amino acid sequence of Tweety gp54 that corresponds to the segment of high mol% G+C in panel a. (c) Sequence of Tweety gp54 showing the locations of repeated sequences. The repeats can be organized as octapeptide repeats (shown as alternating green and red boxes), or as tetrapeptide repeats (shown as alternating darker- and lighter-coloured boxes). Alignments of the sequences of the nucleotide and tetrapeptide repeats are shown in Supplementary Tables S1 and S2 respectively.

Tweety gp54 is a remarkable protein with high sequence similarity (>95 % identity) at both its N- and C-termini to the corresponding parts of Che8 gp57 and PMC gp51 (Fig. 4). The first striking aspect of Tweety gene *54* is the presence of a central core of very high mol% G+C that is prominent within a mol% G+C scan of the entire Tweety genome (Fig. 4a[Fig f4]). Although such a deviation from the average mol% G+C is often indicative of the introduction of DNA elements by horizontal genetic exchange, in this case this seems unlikely. The segment of high mol% G+C corresponds to an apparent expansion of a G+C-rich repeated sequence present in all three related proteins (Supplementary Tables S1 and S2). At the nucleotide level the minimum repeat unit is 12 bp long, of which the first six positions (and their encoded alanine residues) are invariant (Supplementary Table S1). Curiously, positions nine and twelve, which correspond to third codon positions in the utilized reading frame, are also invariant, with greater variation occurring at repeat positions seven (34 Gs, 11 Ts, 3 Cs), eight (45 Gs, 3 As), ten (38 As, 10 Ts) and eleven (38 Gs, 10 As), corresponding to first and second codon positions (Supplementary Table S1). Nevertheless, only two different amino acids are encoded at the fourth residue of the tetrapeptide repeat (serine 38 times, tyrosine 10 times), and three at the third amino acid position (glycine 34 times, tryptophan 11 times, glutamine 3 times) (Supplementary Table S2). This pattern of substitutions within the repeated elements is consistent with selection for variation within this protein.

This repeat sequence is reminiscent of variable region 2 (VR2) in *Bordetella* phage BMP-1 (Liu *et al.*, 2002, 2004), in which a 24 bp element (which includes a 19 bp repeat followed by one of three possible 5 bp spacers) is repeated 9–20 times, depending on the phage isolate, in gene *bbp36*; the role of this variable segment is unknown although it does not appear to reflect changes in host tropism (Liu *et al.*, 2004). While the number of the tetrapeptide repeated segments in mycobacteriophages Tweety gp54, Che8 gp57 and PMC gp51 differs (48 in Tweety, 26 in Che8 and 15 in PMC), these do not simply correspond to the variants observed in different BMP-1 isolates, since the encoded amino acid sequence also differs; in all three phages, the first two positions are invariant alanines, but the composition of the last two positions is distinctly different (Supplementary Table S2). The function of these gene products and the utility of this repeat and its variation is not known, although the finding of similar structures in otherwise unrelated mycobacteriophage and *Bordetella* phages suggests that these may be more widespread throughout phage populations than had been previously recognized. Finally, we note that the entire ORF is absent from mycobacteriophage Llij, even though closely related homologues flanking this gene in Tweety, PMC and Che8 are present (Fig. 3). Presumably, Tweety gp54 is not essential for viral growth, as has been demonstrated for BMP-1 *bbp36* (Liu *et al.*, 2004).

### Tweety integration functions

**Fig. 5. f5:**
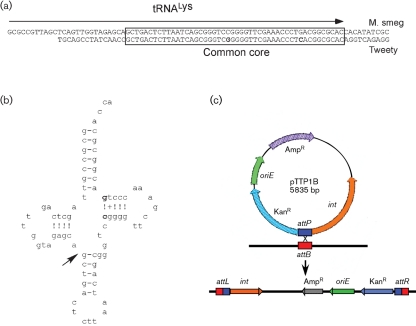
Integration of Tweety into the *M. smegmatis* genome. (a) Sequence alignment of a segment of the Tweety genome immediately upstream of the *int* gene (*43*) (coordinates 32 362–32 290) with part of the *M. smegmatis* genome (coordinates 4 847 908–4 847 991) reveals a common core sequence (boxed); two non-identical positions are shown in bold type. The position of the *M. smegmatis* tRNA^Lys^ gene (Msmeg 4746) is indicated by the arrow. (b) Structure of tRNA^Lys^, with an arrow indicating the position corresponding to the 5′ position of the common core. Positions that change following Tweety integration are shown in bold. (c) Organization of integration-proficient plasmid pTT1B and its integration into the *M. smegmatis* chromosome.

At the 5′ site of the integrase gene (*43*) there is a region of approximately 500 bp that lacks protein-coding potential and is a plausible location for the *attP* site. Comparison of this region with the *M. smegmatis* genome using blastn revealed a short segment of sequence identity (45/47 identical base pairs) that overlaps the 3′ end of a host tRNA^Lys^ gene, a common target for phage integration (Fig. 5a[Fig f5]). This indicates that the *attP* site lies upstream of the Tweety *int* gene and that Tweety integrates at an *attB* site located at coordinates 4 847 939–4 847 986 in the *M. smegmatis* genome. This arrangement also suggests that integration of Tweety results in reconstruction of a hybrid but functional tRNA gene of which the sequence 3′ to the extreme 5′-side of the anticodon stem is phage-derived (Fig. 5b[Fig f5]). Interestingly, the two base differences between Tweety and the *M. smegmatis* genome correspond to the innermost-paired bases in the T*ψ*C loop of the tRNA (Fig. 5b[Fig f5]). Comparison with other mycobacterial genomes shows that this tRNA and the putative *attB* sites are conserved in *M. tuberculosis*, *M. bovis*, *Mycobacterium leprae* and *Mycobacterium avium*. We also note that mycobacteriophages Che8, Llij and PMC contain near-identical integrases and putative *attP* sites, and probably integrate at the same chromosomal location. Che9d has a closely related integrase (39 % amino acid sequence identity) but a different putative *attP* site that we predict recombines at a tRNA^Met^ gene (see below).

### Tweety-based integration-proficient plasmid vectors

The putative *attB* site is at a distinct location from those previously described for phages L5, Ms6 and Bxb1. We therefore reasoned that integration-proficient vectors derived from phage Tweety would integrate independently from those derived from other phages and could thus be used in conjunction with them without interference. Furthermore, the conservation of the host tRNA^Lys^ gene provides a potentially broad host range for integrating plasmids. To construct such vectors, a 1.7 kbp segment of the Tweety genome corresponding to the *int* gene and ∼400 bp of upstream sequences containing the putative *attP* site were PCR amplified and cloned into a plasmid vector containing a kanamycin-resistance gene that cannot replicate in mycobacteria (Fig. 5c[Fig f5]). The two plasmids with the *attP*–*int* segment in either orientation (pTTP1A and pTTP1B) were electroporated into *M. smegmatis* and the numbers of kanamycin-resistant transformants determined (Table 2); both plasmids efficiently transformed *M. smegmatis*, yielding approximately 10^5^ transformants per μg DNA. PCR analysis showed that every transformant tested derived from integration of the plasmid sequences at the predicted *attB* site (data not shown).

To test whether these Tweety integration-proficient vectors are fully compatible with the previously described L5 integration-proficient vectors, we performed co-electroporations with either pTTP1A or pTTP1B DNA and pJV39, an L5 integration vector conferring hygromycin resistance. Co-transformants were readily recovered, indicating that these plasmid integration systems do not interfere with each other (Table 2). We also prepared electrocompetent cells carrying an L5-integration-proficient plasmid vector and showed that pTTP1A efficiently transforms this strain (data not shown). A similar series of experiments were performed using BCG with similar outcomes although the overall transformation frequencies were somewhat lower (Table 2).

The stability of integration-proficient vectors is dependent on the absence of the phage-encoded excise gene. Plasmids pTTP1A and pTTP1B contain no other annotated ORFs other than the integrase gene, so we presume that the putative excise gene is absent. We have not been able to identify any putative excise gene by sequence analysis, although the best candidate is gene *44*, not only because it is adjacent to *int*, but also because there are related copies in phages Che8, PMC and LLij that encode identical integrases (Fig. 3). To test for plasmid stability we grew *M. smegmatis* transformants in the absence of antibiotic selection for approximately 35 generations and then determined the proportion of recovered colonies that had lost the plasmid drug-resistance gene. Under these conditions, we observed that approximately 15 % of cells had lost an L5-derived integrated plasmid (pMH94) whereas only 3.3 % and 7.4 % had lost plasmids pTTP1A and pTTP1B respectively. As noted previously for L5 vectors, the stability of these Tweety vectors could probably be further increased by using a transient integrase-expression system (Hatfull, 2004, 2006).

## DISCUSSION

We have presented here the genome of mycobacteriophage Tweety, a new mycobacteriophage with several interesting and novel features, and its exploitation for the development of integration-proficient vectors that are compatible with those described previously. The Tweety genome is most closely related to those of Che8, PMC and Llij (Hatfull *et al.*, 2006) (Fig. 2), and this close similarity allows more fine-scale conclusions about evolutionary changes than are available from comparisons among more distantly related phages. Only a few of the Tweety gene functions can be readily predicted, although these include several possible restriction endonucleases and several DNA methylases. However, these do not form well-defined restriction–modification cassettes, and combinations of these are not well conserved in the other closely related mycobacteriophages (Fig. 3). For example, homologues of Tweety gp65 are found in Che8, Llij, PMC and Che9d and there are more distant relatives in mycobacteriophages Cjw1 and Wildcat (Hatfull *et al.*, 2006). However, none of these have a closely linked DNA modification function that can be readily recognized.

The presence of a gene encoding a putative family 2 glycosyltransferase (gp104) in the Tweety genome is intriguing since, to our knowledge, this is the first finding of a member of this sequence family of glycosyltransferases in any phage genome. Similar enzymes have been shown previously to be involved in sugar modifications of bacterial cell walls, and gp104 could play a role in phage exclusion similar to the role proposed for the glucosyltransferase in phage SfV (Bastin *et al.*, 1997); however, it is also possible that Tweety gp104 could be involved in DNA modification. Phage T4 and its close relatives encode two glycosyltransferases, and these have long been known to add glucose to hydroxymethyl cytosine residues in phage DNA. If the Tweety enzyme also adds sugars to DNA, this would be an example of analogous but not homologous proteins carrying out the same function in different phages. Other examples include phage lysins, integrases and head-maturation proteases. There do not appear to be any closely related homologues of Tweety gene *104* in any other sequenced mycobacterial genome, and it is therefore unclear from where this gene was acquired. We note that the gene immediately upstream, *103*, has no identifiable homologues in other phage genomes or elsewhere.

Tweety gp54 is unusual with respect to the repeated sequence within the ORF that significantly expands the length of the gene relative to its homologues in phages Che8 (gp57) and PMC (gp51). While the functions of these genes are still unknown, these structures are interesting in their organizational similarity to the VR2 region of *Bordetella* phage BMP-1. The BMP-1 *bbp36* gene that contains VR2 is not essential for phage growth, and we note that Llij does not contain a homologue of Tweety gp54 even though similar flanking genes are present, suggesting that it is not essential for mycobacteriophage growth either. Repeats similar to those in Tweety gp54 are commonly associated with intrinsically unstructured proteins (Tompa, 2003).

The development of integration-proficient vectors with site specificities distinct from those developed previously will provide important tools for constructing recombinant mycobacterial strains. The need for such vectors is illustrated by the development of secondary applications for those derived from phages Ms6 and L5 (Saviola & Bishai, 2004; Vultos *et al.*, 2006), in which either secondary *attB* sites have been introduced or specificities have been altered mutationally, albeit with significant loss of efficiency (Vultos *et al.*, 2006). The Tweety integration vectors not only transform both fast- and slow-growing strains efficiently, but do so in a manner that is fully compatible with integration vectors derived from L5 (Table 2) and Bxb1 (data not shown); it is likely that they are also compatible with Ms6-derived vectors. The Tweety vectors are also maintained with reasonable stability in the absence of drug selection, and somewhat more so than the L5-derived vectors. We have not yet been able to identify the Tweety recombination-directionality factor by sequence comparisons, which is perhaps not surprising given the high sequence divergence of these proteins (Lewis & Hatfull, 2001), although Tweety gp44 remains the best candidate for this function.

While integrase genes can be readily identified in phage genomes, the locations of the *attP* sites require somewhat closer examination. The putative location of Tweety *attP* was indicated by sequence comparison with the *M. smegmatis* genome, and is facilitated by the use of an *attB* site that overlaps a host tRNA gene which is reconstructed following integration. Thus finding a long common core (40 bases or more) that overlaps a host tRNA gene is strongly predictive of the *attB* site location. We have extended this approach to identify potential *attB* sites of other mycobacteriophage integrases in order to identify those that are the best candidates for development of additional integration-proficient vectors with new specificities (Table 3). Using this approach, we predict that phages Che9d, Che9c, Halo and Omega integrate at tRNA^Met^, tRNA^Tyr^, tRNA^Arg^ and tRNA^Leu^ genes respectively, using *attB* sites that are distinct from those of L5, Ms6 and Tweety; three of these phages have conserved *attB* sites in *M. tuberculosis* (Table 3), suggesting that these could be potential broad-host-range integration systems. Interestingly, the Halo integration site is similar to that suggested previously for beta family phages of the Corynebacteria (Cianciotto *et al.*, 1990). This strategy is not applicable for those phages that use serine integrases, although we have identified the *attB* site for the Bxz2 serine integrase, which is located within the Msmeg_5156 ORF, using experimental approaches (Table 3). The Bxz2 *attP* and its *attB* sites share only a 4 bp common core and thus could not simply be identified bioinformatically.

In summary, the genomic analysis of mycobacteriophage Tweety and the development of new integration-proficient vectors further illustrate the general utility of mycobacteriophage studies for mycobacterial genetics. Most of the Tweety genomic functions have yet to be explored or exploited, but this phage promises to have potential utility for understanding other important aspects of mycobacterial and bacteriophage biology and evolution.

## Figures and Tables

**Table 1. t1:** Coordinates and putative functions of Tweety genes

**Gene**	F/R*	**Start position**	**Stop position**	**Mol. mass (kDa)**	**Nearest homologue (% identity)†**	**Function**
1	F	109	564	17.2	PMC gp1 (100 %)	
2	F	587	2224	61.3	Llij gp2 (94 %)	Terminase
3	F	2255	3625	51.2	LLlij gp3 (99 %)	Portal
4	F	3612	4367	27.6	Che8 gp4 (92 %)	
5	F	4393	5043	24.6	Che8 gp5 (97 %)	
6	F	5062	5883	29.2	Llij gp6 (95 %)	Major capsid subunit
7	F	5883	6449	19.8	Che8 gp7 (61 %)	
8	F	6446	6778	11.7	Che8 gp8 (77 %)	
9	F	6781	7110	11.9	Llij gp9 (89 %)	
10	F	7097	7504	14.8	Llij gp10 (66 %)	
11	F	7633	8442	30.1	Llij gp11 (98 %)	Major tail subunit
12	F	8579	9130	20.6	Llij gp12 (85 %)	
13	F	9150	9515	14.0	Llij gp13 (96 %)	
14	F	9534	13 064	119.8	Che8 gp14 (44 %)	Tapemeasure protein
15	F	13 065	14 774	64.1	Llij gp15 (95 %)	
16	F	14 860	16 569	64.2	Che8 gp16 (98 %)	
17	F	16 606	17 454	29.1	Llij gp17 (97 %)	
18	F	17 451	19 988	88.7	Llij gp18 (81 %)	
19	F	19 985	21 871	67.1	Llij gp19 (89 %)	Carboxypeptidase
20	F	21 868	22 116	9.0	Llij gp20 (100 %)	
21	F	22 143	23 033	30.4	Llij gp20 (81 %)	
22	F	23 051	23 392	11.2	Llij gp21 (75 %)	
23	F	23 415	23 669	9.1	Che8 gp23 (95 %)	
24	F	23 682	24 320	21.9	Llij gp23 (90 %)	
25	F	24 317	25 297	30.7	Llij gp24 (71 %)	
26	R	25 301	25 492	7.1	Omega gp45 (100 %)	
27	F	25 642	25 926	9.8	Llij gp26 (78 %)	
28	F	26 076	26 228	5.5	Che9d gp32 (100 %)	
29	F	26 263	26 535	9.8	Llij gp29 (97 %)	
30	F	26 532	27 743	45.4	Ms6 gp2 (94 %)	Lysin A
31	F	27 740	28 741	37.2	Ms6 gp3 (96 %)	Lysin B
32	F	28 751	28 984	8.0	Ms6 gp4 (80 %)	Holin
33	F	28 981	29 355	14.3	Ms6 gp5 (73 %)	
34	F	29 380	29 613	8.7	Llij gp34 (92 %)	
35	F	29 600	30 421	31.3	Llij gp35 (99 %)	DNA polymerase III ε subunit?
36	F	30 508	30 729	8.0	Llij gp36 (98 %)	
37	F	30 722	30 814	3.5	Che8 gp39 (100 %)	
38	R	30 923	31 063	5.8	Che8 gp42 (75 %)	
39	R	31 050	31 391	12.9	Llij gp38 (100 %)	
40	R	31 391	31 600	7.1	Llij gp39 (100 %)	
41	R	31 640	31 879	9.2	Omega gp119 (88 %)	
42	R	31 879	32 046	6.4	Omega gp118 (100 %)	
43	F	32 478	33 767	48.9	Llij gp40 (100 %)	Integrase
44	R	33 843	34 301	16.1	PMC gp39 (100 %)	
45	R	34 564	35 097	19.9	Llij gp42 (63 %)	
46	F	35 251	35 556	11.7	M. therm. hyp.‡ (41 %)	
47	F	35 615	36 619	37.9	PMC gp42 (95 %)	Antirepressor?
48	F	36 616	36 831	7.9	PMC gp43 (100 %)	
49	F	36 917	37 114	7.6	Che8 gp52 (100 %)	
50	F	37 155	37 592	16.8	Che9d gp57 (58 %)	
51	F	37 589	37 777	7.0	Che9d gp58 (70 %)	
52	F	37 774	38 004	8.8	Che8 gp55 (72 %)	
53	F	38 001	38 609	22.8	Che8 gp56 (38 %)	
54	F	38 644	39 768	35.9	Che8 gp57 (94 %)	
55	F	39 847	40 128	10.9	Che8 gp58 (71 %)	
56	F	40 125	40 472	12.7	PMC gp53 (94 %)	*M. tuberculosis* secreted protein?
57	F	40 472	40 873	15.8	PMC gp54 (55 %)	WhiB
58	F	40 870	41 370	19.0	PMC gp55 (71 %)	
59	F	41 367	41 732	13.7	PMC gp57 (63 %)	
60	F	41 732	41 887	6.4	Llij gp57 (86 %)	
61	F	41 884	42 114	8.4	Llij gp58 (74 %)	
62	F	42 111	42 257	5.5	Che9d gp71 (91 %)	
63	F	42 283	42 543	9.8	PMC gp60 (84 %)	
64	F	42 540	43 118	20.8	Llij gp60 (94 %)	
65	F	43 115	43 471	13.7	Llij gp61 (94 %)	Endonuclease
66	F	43 468	44 124	23.9	Che9d gp77 (85 %)	DNA methylase
67	F	44 108	44 341	8.5	Che8 gp72 (100 %)	
68	F	44 341	44 487	5.5	Che8 gp73 (100 %)	
69	F	44 484	45 269	28	Corndog gp7 (56 %)	DNA methylase
70	F	45 248	45 598	12.8	PMC gp78 (84 %)	
71	F	45 591	45 827	8.8	NDM	
72	F	45 824	46 936	38.8	Corndog gp7 (57 %)	DNA methylase
73	F	47 076	47 234	6.2	Llij gp67 (90 %)	
74	F	47 222	47 596	14.3	NDM	
75	F	47 750	48 310	21.5	D. rad. TerF§ (46 %)	HNH endonuclease
76	F	48 310	48 573	9.5	Llij gp70 (88 %)	
77	F	48 570	48 770	7.6	NDM	
78	F	48 767	48 961	7.1	PMC gp73 (96 %)	
79	F	48 958	49 341	13.5	Omega gp81 (100 %)	
80	F	49 334	49 522	7.6	Catera gp14 (76 %)	
81	F	49 480	50 028	20.5	NDM	
82	F	50 025	50 309	10.8	Llij gp77 (100 %)	
83	F	50 302	50 490	7.0	Llij gp78 (78 %)	
84	F	50 560	50 931	14.3	Che8 gp89 (89 %)	
85	F	50 918	51 091	6.5	NDM	
86	F	51 088	51 372	10.4	Omega gp77 (88 %)	
87	F	51 494	51 688	7.5	Che8 gp91 (95 %)	
88	F	51 661	51 951	11.5	NDM	
89	F	51 948	52 127	6.9	Llij gp85 (85 %)	
90	F	52 127	52 264	5.1	PMC gp86 (100 %)	
91	F	52 261	52 434	6.8	PMC gp87 (100 %)	
92	F	52 487	52 654	6.3	PMC gp88 (53 %)	
93	F	52 651	52 914	10.0	Che9d gp10 (51 %)	
94	F	52 911	53 552	24.8	PMC gp92 (81 %)	
95	F	53 662	53 886	8.0	PMC gp93 (97 %)	
96	F	53 883	54 047	6.2	PMC gp94 (96 %)	
97	F	54 054	54 290	8.9	PMC gp95 (97 %)	
98	F	54 235	54 402	6.8	PMC gp96 (69 %)	
99	F	54 399	54 800	14.6	PMC gp97 (84 %)	
100	F	54 797	55 039	8.8	PMC gp98 (64 %)	
101	F	55 032	55 193	6.4	Che8 gp107 (96 %)	
102	F	55 234	55 737	19.1	Omega gp2 (40 %)	Ser/Thr protein kinase?
103	F	55 743	56 279	19.8	NDM	
104	F	56 284	56 958	25.3	D. desul∥ Dde043 (30 %)	Glycosyltransferase?
105	F	57 039	57 245	7.9	Llij gp97 (92 %)	
106	F	57 242	57 367	4.5	NDM	
107	F	57 393	57 602	7.7	NDM	
108	F	57 599	58 225	23.6	PMC gp103 (89 %)	
109	F	58 225	58 551	12.1	PMC gp104 (85 %)	HNH endonuclease

*Direction of transcription, F, forward (leftwards); R, reverse (rightwards). †NDM, no database match. ‡M. therm hyp. *Mycobacterium thermoresistible* hypothetical protein. §D. rad., *Deinococcus radiodurans*. ∥D. desul *Desulfovibrio desulfuricans*.

**Table 2. t2:** Transformation of *M. smegmatis* and BCG by Tweety and L5 integration-proficient vectors

**Plasmid(s)**	**Features**	**Host**	**Transformants [c.f.u. (μg DNA)^−1^]**		
			**Kan^R^**	**Hyg^R^**	**Kan^R^/Hyg^R^**
pTTP1A	Tweety *attP*–*int*; Kan^R^	*M. smegmatis*	2×10^5^	–	–
		BCG	4×10^5^	–	–
pTTP1B	Tweety *attP*–*int*; Kan^R^	*M. smegmatis*	1×10^5^	–	–
		BCG	3×10^5^	–	–
pMH94	L5 *attP*–*int*; Kan^R^	*M. smegmatis*	2×10^5^	–	–
		BCG	2×10^5^	–	–
pJV39	L5 *attP*–*int*; Hyg^R^	*M. smegmatis*	–	3×10^5^	–
		BCG	–	4×10^5^	–
pTTP1A+pJV39	Tweety *attP*–*int*; Kan^R^+L5 *attP*–*int*; Hyg^R^	*M. smegmatis*	2×10^4^	6×10^4^	2×10^3^
		BCG	2×10^4^	1×10^4^	2×10^3^
pTTP1B+pJV39	Tweety *attP*–*int*; Kan^R^+L5 *attP*–*int*; Hyg^R^	*M. smegmatis*	2×10^4^	4×10^4^	8×10^3^
		BCG	2×10^4^	7×10^3^	2×10^3^

**Table 3. t3:** Mycobacteriophage integration systems and putative integration sites

**Phage(s)**	**Integrase type**	***attB* location**	***M. smegmatis* coordinates**	***M. tuberculosis*? (bp identity)**
L5, D29, Che12	Tyr	tRNA^Gly^	4 764 522–4 764 564	Yes (42/43)
Tweety, Che8, Llij, PMC	Tyr	tRNA^Lys^	4 847 934–4 847 981	Yes (46/48)
Ms6	Tyr	tRNA^Ala^	2 213 189–2 213 214	Yes (26/26)
Che9d	Tyr	tRNA^Met^	4 532 823–4 532 867	Yes? (42/45)
Halo	Tyr	tRNA^Arg^	6 410 365–6 410 399	No
Omega	Tyr	tRNA^Leu^	3 328 692–3 328 735	Yes? (39/42)
Che9c	Tyr	tRNA^Tyr^	1 228 421–1 228 480	Yes? (53/57)
CJW1, 244	Tyr	?	?	?
Bxb1, U2, Bethlehem	Ser	*groEL1*		No
Bxz2	Ser	Msmeg_5156	5 259 344–5 259 347	No

## Abbreviations

- BCG, bacille Calmette–Guérin
